# Longitudinal Trends in In-Patient Antibiotic Consumption According to the WHO Access, Watch, Reserve (AWaRe) Antibiotic Groups and Cost: An Analysis of Data at a National Antimicrobial Consumption Network (NAC-NET) Site in North India over 7 Years (2017–2023)

**DOI:** 10.3390/antibiotics13070673

**Published:** 2024-07-19

**Authors:** Niti Mittal, Ashish Tayal, Suneel Kumar, Reevanshi Dhawan, Nidhi Goel, Rakesh Mittal

**Affiliations:** 1 Department of Pharmacology, Pt. B D Sharma Postgraduate Institute of Medical Sciences, Rohtak 124001, Haryana, India; suneelrohilla.pgims@uhsr.ac.in (S.K.); reevanshi.dhawan@gmail.com (R.D.); drrakeshmittal.pgims@uhsr.ac.in (R.M.); 2 Department of Microbiology, Pt. B D Sharma Postgraduate Institute of Medical Sciences, Rohtak 124001, Haryana, India; agupta90345@gmail.com (A.T.); nidhigoel@uhsr.ac.in (N.G.)

**Keywords:** antibiotic surveillance, antibiotic consumption, longitudinal trends, WHO AWaRe classification, defined daily dose, antibiotic stewardship

## Abstract

(1) Background: Antibiotic surveillance data are crucial to map out strategies to promote their optimal use at hospital and community levels. We conducted a comprehensive analysis of longitudinal trends in antibiotic consumption over 7 years at a core “National Antimicrobial Consumption Network” site in North India. (2) Methods: In-patient antibiotic consumption data (2017–2023) were obtained from the hospital’s central drug store and organised as follows: defined daily dose per 100 bed-days; antibiotic consumption as per the WHO access, watch and reserve classification; trends in overall and different antibiotic classes’ consumption; paediatric formulations of antibiotics; and hospital’s annual expenditure on antibiotics. (3) Results: During the 7-year study period, no significant trend could be observed in the overall antibiotic consumption (average annual percent change, AAPC: 9.22; 95% CI: −16.46, 34.9) and cost (AAPC: 13.55; −13.2, 40.3). There was a higher proportion of the consumption of antibiotics in the “reserve” group from 2021 onwards compared to previous years, but the overall trend over 7 years was not significant (AAPC: 319.75; −137.6, 777.1). Antibiotic combinations, classified under the WHO “not recommended” category, comprised a significant proportion of antibiotics consumed. A remarkably increased consumption of azithromycin and doxycycline was recorded during 2020 and 2021, coinciding with the COVID-19 pandemic. (4) Conclusions: Some recommendations to optimise antibiotic use are promoting the use of narrow spectrum “access” group agents; linking antimicrobial resistance and consumption data to formulate effective therapeutic and prophylactic antibiotic use guidelines; and the adoption of restrictive antibiotic policy.

## 1. Introduction

Antimicrobial resistance (AMR) is a worldwide pandemic posing a serious threat to global public health. The excessive and injudicious use of antimicrobials has long been identified as a major risk factor for the emergence of resistant organisms [1]. A startling 65 percent rise in global antibiotic consumption was reported between 2000 and 2015 [2]. As per estimates, approximately 20–50% of all antibiotic use is incongruous and inappropriate, leading to an increased risk of adverse events, greater medical expenses and escalated rates of the emergence of AMR [3]. 

The analysis of antibiotic consumption patterns in hospitals is an integral component of antibiotic stewardship programmes and helps in mapping out strategies to guide and control antibiotic misuse and narrow down AMR. Extensive surveillance programmes have been conducted across various developed countries to analyse the patterns of antibiotic use and AMR [4,5,6,7,8,9]. However, there is lack of accurate data from developing and lower-middle income countries (LMICs), which have shown a worrying hike in the rates of antibiotic consumption over the past few years [10]. India is the biggest consumer of antibiotics in the world [2]. Between 2000 and 2015, antibiotic consumption in India increased at a growth rate of 103 percent [11]. Some studies have reported the proportions of prescriptions containing antibiotics, which vary from 26% to as high as 80% [12,13]. In a large antibiotic surveillance study in a community conducted across multiple healthcare facilities in South India over 2 years, a 40.9% incidence of antibiotic encounters (prescriptions and dispensations) was reported [14]. There is a need to generate data on antibiotic consumption across different hospital settings in India in order to devise strategies for their optimal use, with the ultimate goal of curbing the menace of AMR. 

The Ministry of Health and Family Welfare, India, started the “National Programme on AMR containment” during their 12th five-year plan in 2013 [15]. In 2017, India further released its “National Action Plan on Antimicrobial Resistance (NAP-AMR)” [16], following the footsteps of the “Global Action Plan on Antimicrobial Resistance (GAP-AMR)” launched by WHO in 2015 [17]. One of the strategic priorities identified under the NAP-AMR is to optimise the use of antimicrobials through their surveillance in healthcare facilities [16]. In India, the National Centre for Disease Control (NCDC), New Delhi, is the focal agency for the execution of the “National Programme on AMR containment” [16]. The NCDC established the “National Antimicrobial Consumption Network (NAC-NET)” as a pivotal measure in coordinating antibiotic surveillance. To date, 36 tertiary healthcare centres attached to medical colleges across 27 states and union territories have been enrolled as NAC-NET sites in a phased manner. As a part of this, data on antibiotic consumption at their respective healthcare facilities are compiled and sent periodically to the NCDC by various NAC-NET sites. A “Core NAC-NET sites” group comprising six sites across different zones of the country has also been identified to endow training to other sites. The present study reports the findings of antibiotic surveillance conducted over seven years at one of the collaborating core sites. Through a comprehensive analysis of longitudinal trends in antibiotic consumption, we aim to identify the potential areas for improvement towards the rational use of these agents. 

## 2. Results

**Figure 1:**
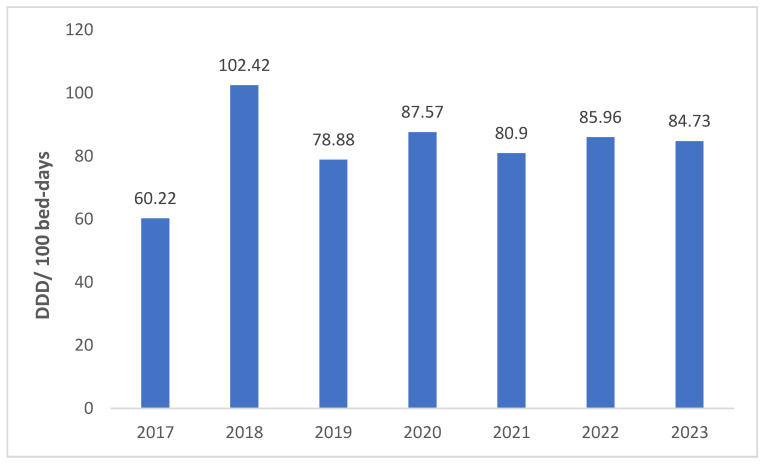
Annual antibiotic consumption (DDD per 100 bed-days) over 7 years (2017–2023). Average annual percent change (AAPC): 9.22%; 95% confidence intervals (CIs): −16.46% to 34.9%).

The total antibiotic consumption in the in-patient departments varied from 60.22 to 102.42 DDD per 100 bed-days during the years 2017–2023, with 2018 and 2017 recording the highest and lowest antibiotic consumption, respectively (Figure 1); in the sensitivity analysis conducted after excluding the 2017 and 2018 consumption data, the average annual percent change was 2.05% (95% CI: −6.02% to 10.13%).

**Figure 2:**
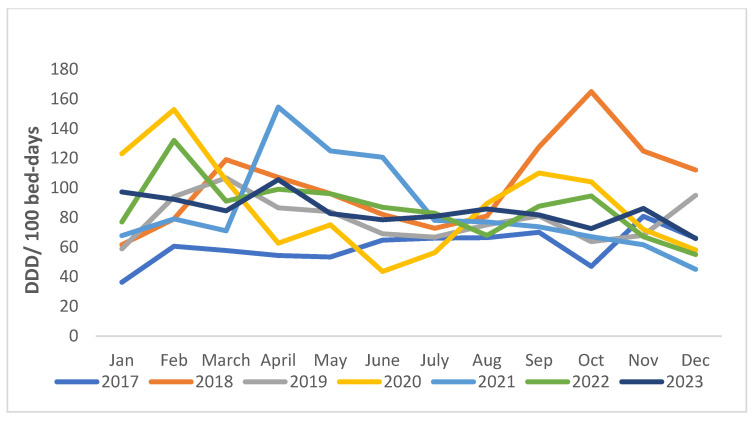
Month-wise trends in antibiotic consumption (DDD per 100 bed-days) over 7 years (2017–2023). Average monthly percent change (AMPC) = 2017: 9.48 (−8.92, 27.87); 2018: 8.75 (−7.33, 24.83); 2019: 6.89 (−7.23, 21); 2020: −1.3 (−20.48, 17.89); 2021: 1.51 (−21.62, 24.63); 2022: 0.35 (−16.3, 17); 2023: −2.45 (−10.95, 6.04).

The month-wise trends in antibiotic consumption showed a surge in consumption during October 2018, February 2020, April and June 2021, and February 2022, while there was a declining trend in antibiotic consumption from April to July 2020 (Figure 2). 

**Figure 3:**
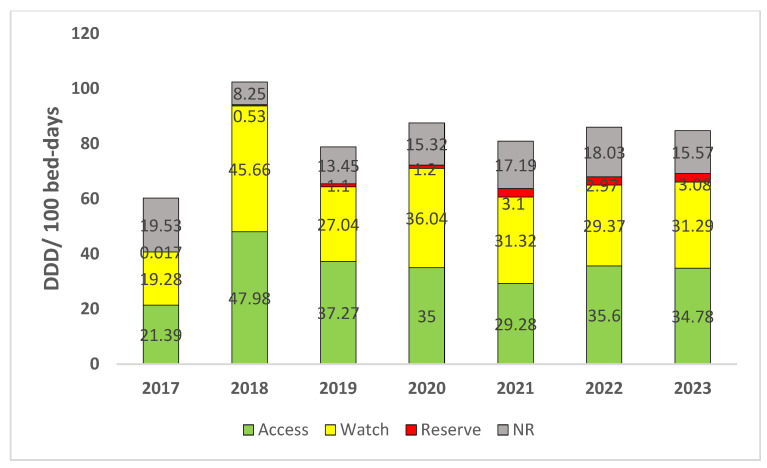
WHO AWaRe category-wise annual antibiotic consumption (DDD per 100 bed-days) over 7 years (2017–2023). (NR: not recommended). Average annual percent change (AAPC) = access: 16.47 (95% CI: −24.24, 57.18); watch: 19.42 (−26.82, 65.66); reserve: 548.69 (−348.66, 1446.04); NR: 3.77 (−25.44, 32.98).

**Figure 4:**
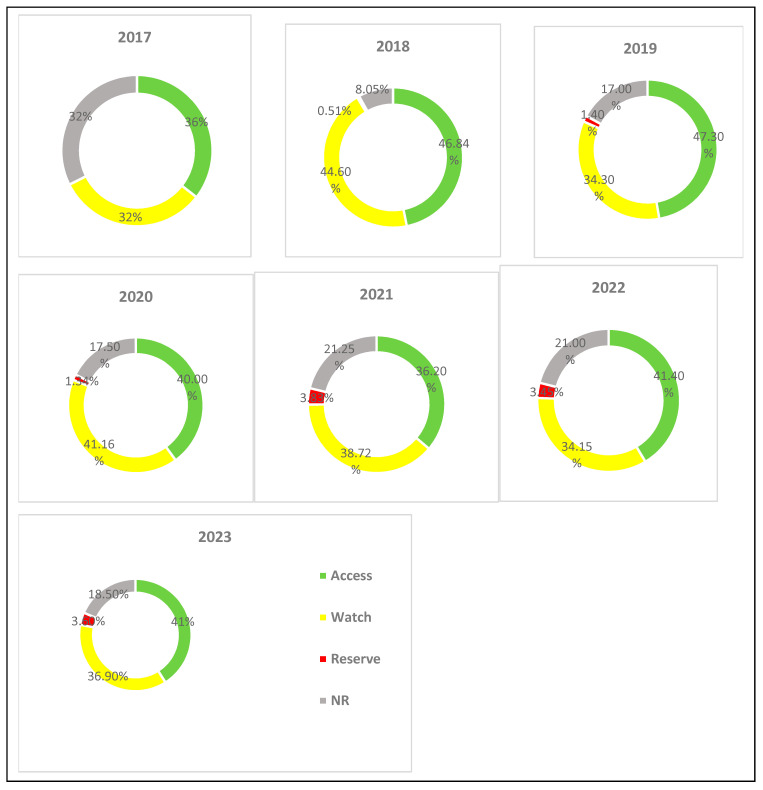
Proportion of annual antibiotic consumption based on WHO AWaRe categories over 7 years (2017–2023). Average annual percent change (AAPC) = access: 3.54 (−9.13, 16.22); watch: 4.42 (−12.42, 21.25); reserve: 319.75 (−137.6, 777.1); NR: 7.85 (−37.08, 52.78).

Overall, the access group of antibiotics comprised the major proportion of antibiotic consumption over 7 years and ranged from 36% (21.39 DDD/100 bed-days) in 2017 to 47% (37.27 DDD/100 bed-days) in 2019. The watch group of antibiotics accounted for more than 30% of the total annual antibiotic consumption throughout the 7-year period, with the maximum proportion recorded in 2018 (44.6%, 45.66 DDD/100 bed-days). Significant reserve category consumption (more than 3%) was observed from 2021 to 2023, while the consumption during the previous 4 years was marginal. Of note, the fixed dose combinations (FDCs) of broad-spectrum agents listed under the “not recommended (NR)” category of the WHO AWaRe classification of antibiotics (ciprofloxacin + tinidazole, ofloxacin + ornidazole, and ceftriaxone/cefoperazone + sulbactam) constituted a sizable proportion of the total annual antibiotic consumption throughout the 7-year observation period, ranging from 8.05% (8.25 DDD/100 bed-days) in 2018 to 32% (19.53 DDD/100 bed-days) in 2017 (Figure 3 and Figure 4). 

Table 1 depicts the annual consumption of various groups of antibiotics according to the WHO ATC/DDD classification. Third-generation cephalosporins followed by penicillin/beta-lactamase inhibitors and macrolides constituted the antibiotic classes with the highest overall consumption. 

**Figure 5:**
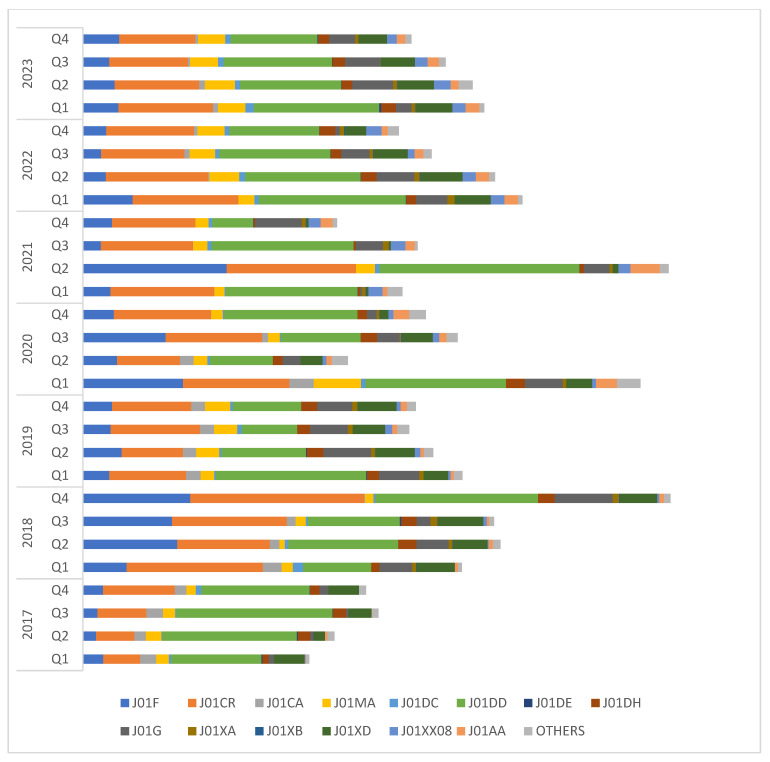
Consumption of different antibiotic classes through different quarters of a year over 7-year period (Q1: January to March, Q2: April to June, Q3: July to September, Q4: October to December). Antibiotics are categorised according to the WHO ATC classification. Data are expressed as DDD/100 bed-days. J01F: macrolides and lincosamides, J01CR: combinations of penicillin including beta-lactamase inhibitors, J01CA: extended spectrum penicillin, J01MA: fluoroquinolones, J01DC: second-generation cephalosporins, J01DD: third-generation cephalosporins, J01DE: fourth-generation cephalosporins, J01DH: carbapenems, J01G: aminoglycosides, J01XA: glycopeptide anti-bacterials, J01XB: polymyxins, J01XD: imidazole derivatives, J01XX08: linezolid, J01AA: tetracyclines. Others: J01EE01 (sulfamethoxazole/trimethoprim), J01RA09 (ofloxacin/ornidazole), J01RA11 (ciprofloxacin/tinidazole).

We studied quarterly trends in the consumption of various classes of antibiotics over the 7-year period (Figure 5 and Table 2). Third-generation cephalosporins including their combinations with beta-lactamase inhibitors (ceftriaxone/cefoperazone–sulbactam; J01DD) followed by penicillin–beta-lactamase inhibitor combinations (J01CR) comprised highly consumed classes of antibiotics consistently over the 7-year period. Intermittent surges in the consumption of macrolides were observed during 2018 (64.5, 60.9 and 73.3 DDD/100 bed-days during Q2, Q3, and Q4, respectively), 2020 (68.5 DDD/100 bed-days in Q1 and 56.5 DDD/100 bed-days in Q3), and 2021 (98.2 DDD/100 bed-days in Q2). An increase in the consumption of tetracyclines (doxycycline), an otherwise less frequently consumed class of antibiotics, was noted during Q1, 2020 (14.36 DDD/100 bed-days), and Q2, 2021 (20 DDD/100 bed-days). 

**Figure 6:**
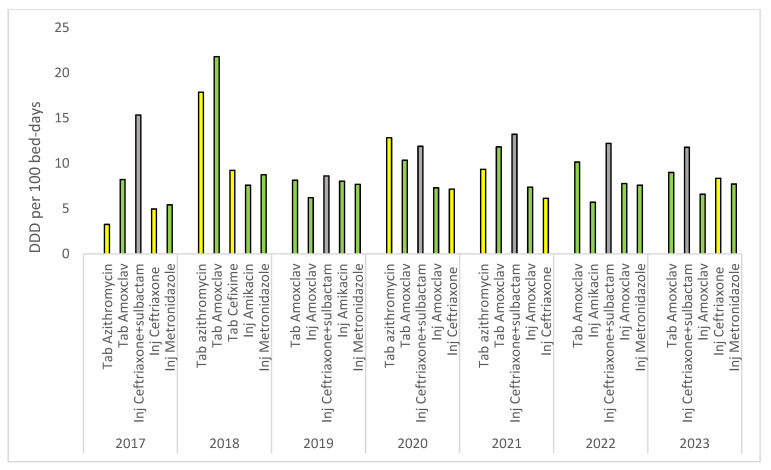
Top five antibiotics consumed year-wise in the hospital during 7 years (2017–2023).

Ceftriaxone–sulbactam injections (belonging to the WHO not-recommended category) followed by amoxicillin–clavulanic acid tablets (access) were the most commonly consumed antibiotics in the hospital during the years 2017, 2019, and 2021–23. In 2018, amoxicillin–clavulanic acid tablets were recorded as the antibiotic with maximum consumption followed by azithromycin tablets (Watch), while in 2020, all these three antibiotics accounted for the major antibiotic consumption, with azithromycin tablets being the top most consumed antibiotic (Figure 6). 

Among the paediatric formulations of antibiotics, amoxicillin (access) and amoxicillin–clavulanate (access) were the most commonly consumed agents over the entire 7-year observation period. There was a surge in the use of azithromycin (watch) during 2018 and 2019; metronidazole (access) and cotrimoxazole (access) were among the other less frequently used antibiotics for the paediatric population. 

The hospital’s annual expenditure on in-patient antibiotics over 7 years ranged from INR (Indian national rupee) 12,677,170 in 2017 to 22,578,148 in 2019. After a sharp rise in annual antibiotic costs in 2018 and 2019, the cost receded in the subsequent 2 years (INR 16,780,275 in 2020 and INR 14,806,518 in 2021), only to witness an increase thereafter (AAPC: 13.55; 95% CI: −13.2, 40.3).

## 3. Discussion

Evaluating the antibiotic consumption trends at local, regional and global levels is essential in order to assess the patterns of their use in clinical practice; this may help in devising strategies to contain their inappropriate consumption, avoiding the development of AMR and measuring the outcomes of stewardship activities in an objective manner. 

In the present study conducted at a tertiary care public hospital in India, during the 7-year period analysed, no significant trend could be observed in the overall antibiotic consumption in DDD per 100 bed-days. We could not find any similar studies from India reporting on timely trends in antibiotic consumption in a hospital set-up, though there are some community-based studies in the literature [14,18,19,20]. Large multicentric studies have analysed the patterns of antibiotic use in upper-middle and high-income countries, some of which have reported upward trends in antibiotic consumption, with the overall growth (over 8- to 10-year periods) ranging from 3.5% to 41% [4,5,6,7,8,9,21,22,23]. Kim et al. reported a stepwise increase in the consumption of broad-spectrum antibiotics and antibiotics for MDR organisms over a 10-year period in a tertiary care hospital in Korea [8]. In Brazil, Castro et al. studied the antibiotic consumption trends across the country from 2013 to 2016 and reported an overall 18% relative growth in antibiotic consumption nationally, with local-level growth ranging from 4% to 85% [24]. In a retrospective analysis of in-patient administrative data drawn from US hospitals, redundant or duplicative antibiotic therapy accounted for more than USD 12 million healthcare costs [9]. A substantial decrease in antimicrobial use in all healthcare institutions was noticed in Israel, following the implementation of a nationwide antimicrobial stewardship programme [25]. In a study conducted by Chen et al. across 89 tertiary general hospitals in China, a decreasing trend in in-patient antibiotic consumption was reported from 2011 to 2015, which was proposed to be related to changes in national and economic policies [26]. 

To date, there seems to be no study from India reporting drug utilisation trends among in-patients based on the WHO AWaRe classification of antibiotics. We observed that the majority of antibiotics consumed belonged to the “access” group, though the proportion was less than the WHO-defined global target (>60% of overall antibiotic use) for healthcare settings [27]; this may partly be explained by the fact that most of the patients already have had encounters with one or more antibiotic before being referred from the periphery to our tertiary care hospital, thus leaving the prescribers with no choice than to resort to limited high-end antibiotics. We observed a considerable increase in the consumption of the reserve group of antibiotics (linezolid and colistin) over the period 2017–2023. Also, despite an observed decrease in the consumption of FDCs of broad-spectrum antibiotics, a high proportion of their use cannot be overlooked considering that the use of this group of agents is neither evidence-based nor recommended in clinical practice by various international guidelines and the WHO [28]. In a point prevalence survey conducted in in-patient wards in our hospital in December 2021, we had reported the WHO AWaRe classification-based percentage distribution of antibiotics as follows: access (44%), watch (41%), reserve (2.7%) and not recommended (12.2%) [29]. The cruciality of favouring narrow-spectrum “access” over the “watch” and “reserve” category groups needs to be realised and enforced as a measure towards combating AMR. 

Some variations in the overall antibiotic consumption and antibiotic classes could be explained by the COVID-19 pandemic. The first wave of COVID-19 spanned from April to September 2020 in India, after which there was a “declining phase” from October to December 2020. In the first wave, nationwide lockdown was enforced by the Government of India from 24 March 2020 to 31 May 2020, during which several restrictions including but not limited to the closure of all non-essential services, etc., were imposed on the entire population. The second COVID wave in India, from March to July 2021, was much more devastating and was associated with higher mortality than the first wave. During the pandemic period, a substantial change in healthcare-seeking behaviours was noted, with fewer people presenting to healthcare facilities with conditions other than COVID-19-associated illnesses [30]. This may account for the recorded decline in overall antibiotic consumption from April to July 2020 and decrease in annual antibiotic expenditure in 2020 and 2021 in our hospital as well. However, the annual antibiotic consumption in 2020 (87.57 DDD per 100 bed-days) was greater than in 2019, i.e., the pre-COVID period (78.88 DDD per 100 bed-days). The increased consumption of macrolides (azithromycin) and doxycycline during 2020 and 2021 coincided with the COVID-19 pandemic, both of which were repurposed for COVID-19 treatment due to their hypothetical anti-inflammatory, anti-viral, immunomodulatory and anti-thrombotic properties [31,32]. Our findings are in agreement with a hospital-based study conducted in central India, where an increase in overall antibiotic consumption, with azithromycin being the most common antibiotic consumed during the COVID-19 pandemic compared to the pre-pandemic period, was reported [33]. Fukushige et al. conducted a systematic review comparing 2019 and 2020 data to study the effect of the COVID-19 pandemic on antibiotic consumption; the authors reported an increase in antibiotic consumption from hospital-based studies in Lebanon, Spain, Italy, the United Kingdom and India [34]. Further, in an interrupted time-series analysis, Sulis et al. reported a significant rise in the sale of azithromycin and doxycycline in India during the COVID-19 pandemic [30], reaffirming the fact that the excess consumption and sale of these antibiotics was the result of a hike in the number of patients seeking healthcare for presumed or confirmed COVID-19 infection in the community as well as in hospitals.

Quarterly trends in antibiotic use were studied for any seasonal variation in the consumption of different antibiotic classes over 12 months. Kotwani et al., in a study assessing the trends of antibiotic consumption among outpatients in New Delhi, a North Indian state, observed a greater use of antibiotics used for acute respiratory infections (penicillin, macrolides, tetracyclines, and cotrimoxazole) in winter months and of anti-diarrhoeal antibiotics (fluoroquinolones) in the humid summer months [18]. We failed to demonstrate any such apparent seasonal trend; this may partly be attributed to the fact that our data are limited to a single public healthcare facility, whereas in the study by Kotwani et al., data were collected through patient exit interviews at private retail pharmacies, public facilities and private clinics. Changes in the resistance patterns over time may also account for such variations, keeping in consideration the time period of this earlier study viz. December 2007 to November 2008.

Our study has some limitations. As noted, we relied mainly on antibiotic dispensing records, which may not have been truly reflective of the amounts consumed such as in cases of changes in treatment before course completion, where, in fact, quantities consumed may have been less than the dispensed; hence, the data presented may be assumed to have over-estimated the consumption of antibiotics. However, since among in-patients, medicines are usually dispensed on a daily basis and procured from central stores as per the demand, we expect not much discrepancy between the amounts dispensed and consumed. Also, this issue is not anticipated to affect the analysis of temporal trends. We gathered hospital-level rather than ward-level data which could have enabled the identification of departments having a relatively higher antibiotic consumption and thus demanding aggressive antibiotic stewardship efforts. Information regarding the indications of antibiotic use (prophylactic or therapeutic) could not be extracted from the data available, making it challenging to differentiate prophylactic and therapeutic antibiotics retrospectively. Also, it would have been interesting to conduct a concurrent analysis of AMR surveillance data which could have helped in establishing any resistance–consumption linkage, in turn providing a strong basis for antibiotic policy; this was, however, beyond the scope of our study. DDD per 100 bed-days is a universal standard for comparing antibiotic consumption among different centres, yet its application has a few limitations; DDD assumes the routine maintenance dose of a drug, so in cases of higher (such as augmented renal clearance, central nervous system infections, critically ill patients, etc.) or lower (compromised renal function, paediatric populations, etc.) than standard dosing, the use of DDD may erroneously estimate the exposure [35]. In fact, days of therapy (DOT, all antibiotics a particular patient receives over a specific time) is being identified as a more clinically relevant indicator and is recommended in some antibiotic stewardship guidelines [36]. Besides this, DDD per 100 bed-days captures the antibiotic pressure at the hospital level without taking into account the selective pressure at the patient level. It is worth noting that in the public sector, an important determinant in the selection of antibiotics is their supply and availability in the hospital’s central store. Previously, we assessed the availability of antimicrobials across public and private sector facilities in our district, including our hospital, and an overview of the findings of the two studies informed us that most of the drugs prescribed were available in the hospital’s pharmacy [37]. Nevertheless, additional data on drugs purchased by patients due to reasons such as non-availability in stock or not included in the hospital formulary would have been a true reflection of antibiotic use. The study data are restricted to a single public tertiary care hospital; hence, the associated drawbacks such as selection bias and limited generalizability need to be considered while interpreting the results. 

Despite limitations, certain strengths of our study need to be mentioned. The present study reports longitudinal trends in in-patient antibiotic consumption in a tertiary care public hospital in North India. Although there are few studies providing a snapshot of antibiotic consumption at local and regional levels from India, all of them used community or outpatient data, with none focussing on antibiotic usage at the hospital level [14,18,19,20]. This is also the first study analysing and reporting comprehensive aggregate data on hospital antibiotic surveillance collected over a period of 7 years as part of the National Antimicrobial Consumption Network (NAC-NET), ours being one of the core NAC-NET sites in India. Antibiotic consumption was also assessed according to the WHO AWaRe classification of antibiotics, which is a globally recommended standard indicator for antibiotic use and antimicrobial stewardship programmes. 

## 4. Materials and Methods

### 4.1. Study Setting

The study site, the Postgraduate Institute of Medical Sciences (PGIMS), Rohtak, is a 2080-bedded public tertiary care hospital catering to patients from Haryana and its adjacent states, with approximately 0.4–0.5 million annual admissions. As one of the sites included under the “National Programme on AMR containment” and one of the six core NAC-NET sites, regular surveillance of antibiotic consumption has been conducted in the hospital since 2017 and the compiled data sent to NCDC periodically.

### 4.2. Data Collection

Antibiotic consumption data are routinely collected by a trained pharmacist recruited at the site under the National Programme on AMR containment. Data on in-patient antibiotic consumption are obtained from a central drug store, while bed occupancy rate data are sourced from the Medical Records Department every month. Consumption estimates are reported in standard units (SUs) defined as the smallest dose of formulation like one tablet or capsule for oral solid dosage formulations and one vial or ampoule for injectable antibiotics. Antibiotics consumed at in-patient facilities only and prescribed through oral and parenteral routes are included in the surveillance data. Antibiotics prescribed by any other route such as topical preparations, eye/ear drops, gels and suppositories, etc., are excluded. 

### 4.3. Data Management and Analysis

Data on antibiotic consumption are recorded in a master sheet along with their WHO AWaRe classification [28]. A csv file is created using the following variables: the name of the antibiotic, pack size, the strength of the drug, the WHO ATC (anatomical, therapeutic and chemical) code and the route of administration. Data, thus entered, were imported to WHO AMC tool 2019 v 1.9.0. [38] and the defined daily doses for each antibiotic calculated. 

Data are subjected to the following analyses:

1. Number of defined daily doses (DDDs): DDD is the assumed average maintenance dose per day for a drug used for its main indication in adults. The number of DDDs is calculated as per the following formula:
  Number of DDDs = Number of items issued × amount of drug per item/WHO DDD measure.
2. The bed occupancy rate (%) is calculated as follows:
  Bed occupancy rate = (Total number of in-patient days for a given period × 100)/(Number of available beds × number of days in that period).
3. DDD/100 bed-days is calculated as follows:
  DDD/100 bed-days = (Number of DDDs × 100)/(Number of beds × number of days in the period × occupancy index).

### 4.4. Measures of Antibiotic Consumption

For the purpose of this study, antibiotic consumption was organised using the following key indicators: (1) DDDs/100 bed-days; (2) antibiotic consumption as per the WHO AWaRe classification; (3) antibiotic consumption according to the WHO ATC/DDD classification; (4) annual and monthly trends in overall antibiotic consumption; (5) quarterly trends in the consumption of different classes of antibiotics; (6) a list of the top 5 antibiotics, defined as the five most commonly consumed antibiotics during each year over the study period; (7) paediatric formulations of antibiotics; and (8) the hospital’s annual expenditure on antibiotics. The expenditure on antibiotics was calculated according to the purchase rate of antibiotics available on the government rate contract, the site being a government tertiary care hospital. 

### 4.5. Statistical Analysis

Various trends in antibiotic consumptions were evaluated as follows:

#### 4.5.1. Average Annual Percent Change (AAPC)

$$Annual\;percent\;change\;(APC)=\frac{Value\;in\;current\;year-Value\;in\;previous\;year}{Value\;in\;previous\;year}\times 100$$

$$AAPC=\frac{Sum\;of\;all\;annual\;percent\;changes\;(APCs)}{N}$$

where N = the total number of periods and equals 7, corresponding to the 7-year study period. 

#### 4.5.2. Average Monthly Percent Change (AMPC)

$$Monthly\;percent\;change\;(MPC)=\frac{Value\;in\;current\;month-Value\;in\;previous\;month}{Value\;in\;previous\;month}\times 100$$

$$AMPC=\frac{Sum\;of\;all\;monthly\;percent\;changes\;(MPCs)}{N}$$

where N = the total number of months and equals 12, corresponding to 12 months in a year.

#### 4.5.3. Average Quarterly Percent Change (AQPC)

$$Quarterly\;percent\;change\;(QPC)=\frac{Value\;in\;current\;quarter-Value\;in\;previous\;quarter}{Value\;in\;previous\;quarter}\times 100$$

$$AQPC=\frac{Sum\;of\;allquarterly\;percent\;changes\;(QPCs)}{N}$$

where N = total number of quarters and equals 4, corresponding to 4 quarters in a year.

All the calculations were performed in Microsoft Excel (version 2109). Moreover, 95 percent confidence intervals for all average percent changes were derived using the “Confidence” function in Excel, with an alpha value of 0.05. 

### 4.6. Ethical and Administrative Approvals

The study was conducted after obtaining ethical and administrative approval from the Biomedical Research Ethics Committee of PGIMS, Rohtak, Haryana (vide letter no. BREC/24/23 dated 24 January 2024) and the Office of the Medical Superintendent, PGIMS, Rohtak, Haryana (vide letter no. PGIMS/Misc/23/8948-51 dated 17 October 2023), respectively.

## 5. Conclusions

Generating antibiotic surveillance data across different settings and regions is crucial to identify the areas for improvement and take steps in the right direction to optimise and rationalise the use of these agents at hospital and community levels. The present study at a tertiary care public hospital, a core NAC-NET site in North India, reported trends in hospital antibiotic consumption over 7 years. From the data gathered, some areas demanding attention in our setting are as follows: decreasing the use of reserve/high-end antibiotics and antibiotic combinations, promoting the use of the “access” group of antibiotics, and linking antimicrobial resistance and consumption data to reinforce and implement an effective antibiotic policy. There is a need for a multidisciplinary therapeutics committee involving infectious disease physicians, microbiologists and pharmacologists, which can formulate strategies to reduce the consumption and cost of antibiotics in hospitals. Some key fundamental steps in this direction could be the adoption of a restrictive antibiotic policy, framing in-patient therapeutic and prophylactic antibiotic guidelines based on local microbial resistance patterns and prescriber education.

## Figures and Tables

**Table 1:** Annual consumption of various WHO ATC/DDD antibiotic groups over 7 years.

Class of Antibiotics	2017	2018	2019	2020	2021	2022	2023	Overall Consumption	AAPC (95% CI)
J01F: Macrolides and lincosamides	46.8	228.7	83.5	169.6	149.4	78.1	89	845.1	63.77 (−61.92, 189.46)
J01CR: Penicillin/beta-lactamase inhibitors	134	353.4	209.5	248	279.2	259.2	228.2	1711.5	22.47 (−31.07, 76.02)
J01CA: Extended spectrum penicillin	38	25.9	38	29.96	0.1	6.5	10.3	148.76	1058.75 (−880.13, 2997.64)
J01MA: Fluoroquinolones	34	24.1	58	57.76	38.6	67.4	77.2	357.06	27.85 (−22.24, 77.94)
J01DC: Second-generation cephalosporins	6.4	12	7.3	6.29	9.05	12.7	16.4	70.14	24.64 (−8.72, 58)
J01DD: Third-generation cephalosporins	334	295.3	245.1	284.3	351.5	316.8	288.1	2115.1	−1.31 (−13.74, 11.11)
J01DE: Fourth-generation cephalosporins	2.12	0.97	1.2	0	0.9	0.96	2.48	8.63	--
J01DH: Carbapenems	28.8	40.1	39.13	37.5	6.8	35.5	33.4	221.23	61.15 (−73.01, 195.32)
J01G: Aminoglycosides	12.76	93.3	109.8	59.75	69.05	69.4	80.2	494.26	105.82 (−85.67, 297.31)
J01XA: Glycopeptide anti-bacterials	0	14.4	12.5	5.17	11.47	13.8	8	65.34	--
J01XB: Polymyxins	0.14	1.6	0.26	1.47	1.42	1.8	1.28	7.97	236.49 (−90.99, 563.98)
J01XD: Imidazole derivatives	65.6	106.2	92.5	59.45	7.69	91.2	92.1	514.74	168.86 (−165.91, 503.62)
J01XX08: Linezolid	0.6	4.63	13	12.93	36.1	33.4	35.7	136.36	171.75 (−21.15, 364.65)
J01AA: Tetracyclines	2.03	9.7	12.3	33.86	37.8	28.5	28.3	152.49	94.38 (−21.1, 209.85)
Others	16.4	15.5	26.5	45.8	21.3	20.3	21.5	167.3	14.34 (−22.04, 50.71)

Data expressed as DDD per 100 bed-days. AAPC: average annual percent change.

**Table 2:** Quarterly trends in consumption of different antibiotic classes over 7 year period.

Class of Antibiotics	2017	2018	2019	2020	2021	2022	2023
J01F	4.47 (−32.09, 41.02)	43.26 (−18.94, 105.46)	8.06 (−29.02, 45.14)	4.38 (−111.97, 120.72)	131.57 (−123.68, 386.82)	−15.27 (−56.16, 25.62)	3.54 (−26.2, 33.28)
J01CR	26 (5.44, 46.57)	14.58 (−27.18, 56.34)	4.59 (−30.16, 39.34)	4.44 (−41.86, 50.74)	−4.58 (−31.11, 21.95)	−5.45 (−17.31, 6.40)	−6.93 (−10.45, −3.41)
J01CA	−4.15 (−47.35, 39.05)	−49.66 (−90.8, −8.51)	−1.81 (−9.98, 6.36)	−66.49 (−94.39, −38.58)	--	--	−5.19 (−58.26, 47.88)
J01MA	−6.97 (−33.61, 19.67)	5.21 (−63.58, 74)	26.17 (−9.34, 61.68)	−28.72 (−65.15, 7.72)	17.42 (−39.98, 74.82)	25.65 (−26.03, 77.34)	0.11 (−9.1, 9.32)
J01DC	105.24 (−74.54, 285.02)	−35.27 (−68.1, −2.44)	34.72 (−32.88, 102.32)	−27.15 (−66.19, 11.89)	94.21 (−80.46, 268.87)	8.46 (−24.98, 41.89)	−10.93 (−36.4, 14.54)
J01DD	11.42 (−27.65, 50.5)	40.53 (−8.43, 89.48)	−18.35 (−53.68, 16.97)	12.96 (−48.23, 74.16)	−16.14 (−77.28, 45.01)	−14.84 (−24.08, −5.6)	−10.73 (−25.99, 4.53)
J01DE	−15.01 (−53.29, 23.27)	--	--	--	38.89 (−98.09, 175.87)	13.33 (−65.88, 92.54)	−2.22 (−79.31, 74.87)
J01DH	24.72 (−37.74, 87.19)	38.52 (−34.22, 111.26)	13.58 (−17.62, 44.77)	−7.86 (−70.74, 55.02)	10.79 (−77.87, 99.46)	25.4 (−25.5, 76.31)	−7.05 (−28.55, 14.45)
J01G	89.33 (−124.45, 303.12)	87.32 (−113.04, 287.69)	−3.08 (−22.77, 16.61)	−27.51 (−74.22, 19.2)	499.66 (−283.53, 1282.86)	−29.21 (−81.4, 22.97)	39.96 (−61.94, 141.87)
J01XA	--	23.74 (−22.66, 70.13)	6.82 (−3.64, 17.28)	301.26 (−215.9, 818.42)	14.97 (−57.24, 87.17)	−13.53 (−42.71, 15.64)	185.07 (−198.58, 568.72)
J01XB	--	44.44 (−30.99, 119.88)	--	--	--	--	−24.47 (−91.03, 42.08)
J01XD	23.23 (−57.21, 103.68)	0.96 (−24.74, 26.65)	20.67 (−16.74, 58.08)	−16.28 (−71.69, 39.14)	62.89 (−103.06, 228.83)	−13.35 (−39.91, 13.22)	−7.63 (−14.73, −0.54)
J01XX08	--	177.32 (−5.6, 360.24)	35.6 (−45.37, 116.57)	16.37 (−27.86, 60.6)	−3.84 (−25.76, 18.08)	23.42 (−62.44, 109.29)	−6.66 (−34.97, 21.65)
J01AA	80.49 (−219, 379.97)	33.39 (−38.25, 105.04)	29.29 (20.13, 38.44)	25.63 (−67.93, 119.19)	155.91 (−145.17, 456.98)	−21.6 (−37.6, −5.6)	−8.34 (−53.49, 37.11)
0thers	36.37 (−25.34, 98.08)	32.26 (−34.64, 99.17)	2.78 (−25.21, 30.76)	−4.49 (−49.62, 40.65)	−19.47 (−79.68, 40.74)	35.75 (32.14, 39.37)	46.6 (−83.1, 176.3)

Data represent the AQPC, i.e., average quarterly percent change (95% confidence intervals). J01F: macrolides and lincosamides, J01CR: combinations of penicillin including beta-lactamase inhibitors, J01CA: extended spectrum penicillin, J01MA: fluoroquinolones, J01DC: second-generation cephalosporins, J01DD: third-generation cephalosporins, J01DE: fourth-generation cephalosporins, J01DH: carbapenems, J01G: aminoglycosides, J01XA: glycopeptide anti-bacterials, J01XB: polymyxins, J01XD: imidazole derivatives, J01XX08: linezolid, J01AA: tetracyclines. Others: J01EE01 (sulfamethoxazole/trimethoprim), J01RA09 (ofloxacin/ornidazole), J01RA11 (ciprofloxacin/tinidazole).

## Data Availability

The data presented in this study are available on request from the corresponding author. The data are not publicly available due to privacy concerns.
